# The Sigma-2 Receptor and Progesterone Receptor Membrane Component 1 are Different Binding Sites Derived From Independent Genes

**DOI:** 10.1016/j.ebiom.2015.10.017

**Published:** 2015-10-19

**Authors:** Uyen B. Chu, Timur A. Mavlyutov, Ming-Liang Chu, Huan Yang, Amanda Schulman, Christophe Mesangeau, Christopher R. McCurdy, Lian-Wang Guo, Arnold E. Ruoho

**Affiliations:** a Department of Neuroscience, University of Wisconsin School of Medicine and Public Health, Madison, WI, United States; b Department of Surgery, University of Wisconsin School of Medicine and Public Health, Madison, WI, United States; c McPherson Eye Research Institute, University of Wisconsin School of Medicine and Public Health, Madison, WI, United States; d Department of BioMolecular Sciences, School of Pharmacy, University of Mississippi, United States

**Keywords:** Progesterone receptor membrane component-1 (PGRMC1), Sigma-2 receptor (S2R), [^3^H]-1,3-Di-o-tolylguanidine ([^3^H]-DTG), [^125^I]-Iodoazido-fenpropimorph ([^125^I]-IAF), Clustered regularly interspaced short palindromic repeats (CRISPR)/Cas9 knockout

## Abstract

The sigma-2 receptor (S2R) is a potential therapeutic target for cancer and neuronal diseases. However, the identity of the S2R has remained a matter of debate. Historically, the S2R has been defined as (1) a binding site with high affinity to 1,3-di-o-tolylguanidine (DTG) and haloperidol but not to the selective sigma-1 receptor ligand (+)-pentazocine, and (2) a protein of 18–21 kDa, as shown by specific photolabeling with [^3^H]-Azido-DTG and [^125^I]-iodoazido-fenpropimorph ([^125^I]-IAF). Recently, the progesterone receptor membrane component 1 (PGRMC1), a 25 kDa protein, was reported to be the S2R (*Nature Communications*, 2011, 2:380). To confirm this identification, we created PGRMC1 knockout NSC34 cell lines using the CRISPR/Cas9 technology. We found that in NSC34 cells devoid of or overexpressing PGRMC1, the maximum [^3^H]-DTG binding to the S2R (B_max_) as well as the DTG-protectable [^125^I]-IAF photolabeling of the S2R were similar to those of wild-type control cells. Furthermore, the affinities of DTG and haloperidol for PGRMC1 (K_I_ = 472 μM and 350 μM, respectively), as determined in competition with [^3^H]-progesterone, were more than 3 orders of magnitude lower than those reported for the S2R (20–80 nM). These results clarify that PGRMC1 and the S2R are distinct binding sites expressed by different genes.

## Highlights

• The sigma-2 receptor is an important drug target but its molecular identity has remained a hot topic of debate.
• PGRMC1 has recently been reported to be the sigma-2 binding site (*Nature Communications*, 2011, 2:380).
• Our data clarify that PGRMC1 and the sigma-2 receptor are distinct binding sites expressed by different genes.

The sigma-2 receptor (S2R) is a potential important therapeutic target for cancer and neuronal diseases, yet its gene identity is a long-held mystery. While a recent prominent report concluded that a progesterone-binding protein (PGRMC1) is the sigma-2 receptor, the critical defining evidence was missing. We re-tested this conclusion using a genome-editing technology combined with chemical biology and pharmacological determinations. The unambiguous results indicate that PGRMC1 is not the originally defined true sigma-2 receptor. This study may benefit public health by guiding future discovery of the true identity of the sigma-2 drug binding site.

## 1 Introduction

Recently there has been a resurgence of interest in sigma receptors. Sigma binding sites were classified into Sigma-1 (S1R) and Sigma-2 (S2R) receptors based on earlier pharmacological studies (Bowen et al., 1989, Hellewell and Bowen, 1990, Hellewell et al., 1994, Quirion et al., 1992). While the S1R was cloned and has been shown to play important roles in a plethora of tissues and cells, the molecular identity of the S2R remains obscure (for review, see Matsumoto et al. (2014)). Pharmacological and chemical biology studies have revealed that the S2R is highly expressed in tumors and S2R ligands have shown anti-cancer effects (Crawford and Bowen, 2002, Hornick et al., 2010, Kashiwagi et al., 2009, Vilner and Bowen, 2000, Vilner et al., 1995, Wheeler et al., 2000). In addition, the S2R is also implicated to play an important role in the nervous system (Vilner and Bowen, 1993, Vilner and Bowen, 2000). However, the lack of a known S2R amino acid sequence is greatly impeding progress in identifying S2R's biological functions as well as potential therapeutic methods targeting this receptor. It is thus imperative to discover its genetic identity.

Sigma receptors are arguably the most mysterious signaling proteins. While the S1R was mischaracterized as an opioid receptor (Martin et al., 1976) until its gene sequence became available (Hanner et al., 1996), the S2R has evaded many attempts to decipher its genetic coding. For example, mass spectrometry data suggested histones or associated proteins as S2R ligand binding proteins (Colabufo et al., 2006), but they differ from the S2R in membrane association. The difficulty in unveiling the S2R molecular identity may stem from the fact that it resides in lipid rafts and is not readily detergent-extractable without compromising its functional integrity (Gebreselassie and Bowen, 2004). Moreover, the abundance of this protein in membranes prepared from mammalian tissues is extremely low (e.g*.* 0.1 μg/mg total protein, Ruoho et al*.*, unpublished data).

The S2R has been historically defined as a binding site with high-affinity (20–80 nM) for DTG (1,3-di-o-tolylguanidine) and haloperidol (Vilner et al., 1995, Hellewell et al., 1994, Matsumoto et al., 2014). Its molecular size was shown to be 18–21 kDa by [^3^H]-Azido-DTG photoaffinity labeling (Hellewell and Bowen, 1990, Bowen et al., 1989). To readily “visualize” the S2R, our group developed a highly sensitive photoaffinity probe, [^125^I]-iodoazido-fenpropimorph ([^125^I]-IAF) (Pal et al., 2007). In accordance with the [^3^H]-Azido-DTG photolabeling data (Hellewell and Bowen, 1990, Hellewell et al., 1994), the [^125^I]-IAF photolabeled S2R band appears at ~ 18 kDa on a SDS gel. Importantly, this S2R band is protected by DTG and haloperidol but not (+)-pentazocine, a specific S1R ligand. Using this chemical biology method we have been able to discriminatively detect the S2R and S1R and their specificity for novel sigma receptor ligands (Pal et al., 2007, Fontanilla et al., 2009, Fontanilla et al., 2008).

A recent finding by Xu et al*.* identified progesterone receptor membrane component-1 (PGRMC1) as the S2R (Xu et al., 2011). While this discovery is expected to open broad-spectrum opportunities in research and/or the development of therapeutic interventions given the functional importance of the S2R, critical questions remain. First, the apparent molecular weight of PGRMC1 on SDS gels is 25 kDa rather than 18–21 kDa as previously reported for the S2R (Pal et al., 2007, Hellewell and Bowen, 1990). Our earlier studies using [^125^I]-IAF showed only two DTG (or haloperidol)-protectable photolabeled bands on the SDS gel, namely the S2R and the S1R. We did not detect an ~ 25-kDa band that is consistent with PGRMC1 (Ruoho et al., 2013, Fontanilla et al., 2009, Pal et al., 2007), not even with PGRMC1 overexpressed (Chu et al., 2015, Ruoho et al., 2013). Second and more importantly, a high-affinity (20–80 nM) binding with DTG or haloperidol is the signature of the S2R, but the DTG (or haloperidol) binding affinity for PGRMC1 has never been reported. It is worth noting that in spite of this ambiguity, perception of PGRMC1 as the S2R has become increasingly accepted among researchers working on PGRMC1 (Mir et al., 2013, Bali et al., 2013, Izzo et al., 2014). It is therefore important to clarify whether PGRMC1 is truly the S2R. In this study, we aimed to explicitly answer two key questions: 1) is the S2R a splice variant of PGRMC1 and 2) does PGRMC1 bind with high affinity to DTG and haloperidol — the signature of the S2R.

## 2 Methods

### 2.1 Reagents

CM compounds were synthesized in the McCurdy Lab (University of Mississippi, University, MS). [^3^H]-(+)-1,3-ditolyl guanidine (DTG) and [^3^H]-progesterone were obtained from PerkinElmer, (Waltham, MA). Nonradioactive DTG, haloperidol, (+)-pentazocine and progesterone were obtained from Sigma-Aldrich (St. Louis, MO). Rabbit anti-PGRMC1 antibody was purchased from Proteintech (Chicago, IL; Cat. No. 12990-1-AP). PGRMC1 cDNA was obtained from Origene (Rockville, MD). All other reagents were from Sigma-Aldrich or Thermo-Fisher unless specifically stated.

### 2.2 Cell Culture

NSC34 cells were grown in 15 cm cell culture dishes in 50%/50% DMEM/F12 supplemented with 10% fetal bovine serum and 1 × penicillin/streptomycin (final 100 μg/ml). PC12 cells were grown in RPMI 1640 (Mediatech, Manassas, VA) supplemented with 10% heat-inactivated horse serum (Corning, Manassas, VA), 5% fetal bovine serum, and 1 × penicillin/streptomycin.

### 2.3 Generation of CRISPR/Cas9 Constructs for PGRMC1 Knockout

For knocking out PGRMC1 using the clustered regularly interspaced short palindromic repeats (CRISPR)/Cas9 technology, sgRNAs targeting PGRMC1 exon-1 were cloned into a Cas9-expressing lentiviral transfer vector (lentiCRISPRv2, Cat No. 52961, Addgene, Cambridge, MA) following the methods of the Feng Zhang laboratory (Sanjana et al., 2014). Shown below are the two oligonucleotides from the sense strands (clones 38 and 207) that were used for sgRNAs to target PGRMC1 exon-1.

#38: 5′-ACCCGAGCGAGCTAGAGGGC-3′.

#207: 5′-CAAGCGGCGCGACTTCACCC-3′.

Oligonucleotides for the sgRNA guide sequence (both sense and antisense strands) were synthesized at the Biotechnology Center, University of Wisconsin — Madison. sgRNA oligos were first phosphorylated using polynucleotide kinase (Life Technologies, Grand Island, NY) at 37 °C for 30 min and then annealed (sense to antisense strands) at 95 °C for 5 min and then ramp down to 25 °C at 5 °C/min. To prepare LentiCRISPRv2 for insertion of oligos, LentiCRISPRv2 was digested and dephosphorylated with FastDigest BsmBI and FastAP (Life Technologies, Grand Island, NY) at 37 °C for 2 h. To insert the sgRNA annealed oligos into digested/dephosphorylated LentiCRISPRv2, oligos were ligated into the plasmid using T7 ligase (Enzymatics, Beverly, MA) at 25 °C for 5 min. Cloned transfer plasmids were amplified using an endotoxin-free midi-prep kit (Qiagen, Valencia, CA).

### 2.4 Packaging of Lentiviruses

To prepare lentiviruses for PGRMC1 knockout, each of the two LentiCRISPRv2–sgRNA PGRMC1 (#38 and #207) transfer plasmids was co-transfected with packaging plasmids pMD2.G and psPAX2 (Addgene plasmids 12259 and 12260), as described previously (Zufferey et al., 1998). Briefly, HEK293T cells (ATCC CRL-3216) at 80% confluency in a 35-mm dish were transfected with 2 μg of the transfer plasmid, 0.2 μg of pMD2.G, and 0.75 μg of psPAX2, in DMEM (Life Technologies, Grand Island, NY) added with 10% fetal bovine serum (Hyclone, Logan, Utah) and 4 μl of JetPrime transfection reagent (POLYPLUS-TRANSFECTION Inc., NY). After 72 h, the supernatant was harvested and centrifuged at 3000 rpm at 4 °C for 10 min to pellet cell debris. The virus-containing supernatant was filtered through a 0.45 μm low protein binding membrane (Millipore, Billerica, MA) and used immediately for creating PGRMC1 knockout cell lines.

### 2.5 Lentiviral Transduction for PGRMC1 Knockout

For viral transduction to knockout PGRMC1, ~ 2.5 × 10^4^ NSC34 cells were incubated with the foregoing filtered lentivirus-containing supernatant. After 3 days of transduction, puromycin was added (final 3 μg/ml) to screen sgRNA/Cas9 positive cells. Two weeks later the cell culture was expanded to three 35-mm dishes. In order to assess the efficiency of sgRNA-guided Cas9 cutting in the PGRMC1 genomic sequence (exon-1), genomic DNA was extracted for PCR amplification of the specific region including the sgRNA/Cas9 excision site. Forward primer: gcggaggaagcggactgttc; reverse primer: agcgggccgggggcacgagg. PCR products were digested with 1 μl T7 Endonuclease I (New England Biolabs Inc., MA) for 2 h at room temperature, and then subjected to electrophoresis in a 1.5% agarose gel. PGRMC1 protein knockout was confirmed by Western blotting using a PGRMC1 specific antibody.

### 2.6 Construct of pCI/Neo-PGRMC1-3xHA for PGRMC1 Overexpression

Full-length PGRMC1 was engineered to contain a C-terminal 3 × HA tag through polymerase chain reaction (PCR)-engineered MluI restriction enzyme site that replaced the native stop codon. PCR amplified PGRMC1 was placed between NheI and MluI restriction enzyme sites and the 3 × HA tag was inserted between MluI and NotI restriction enzyme sites on a pCI/neo vector (Promega, Madison, WI). The pCI/Neo-PGRMC1-3 × HA clone was confirmed by sequencing at the Biotechnology Center, University of Wisconsin — Madison. A pCI/neo vector without the PGRMC1 gene served as control.

### 2.7 Plasmid Transfection

To overexpress PGRMC1, NSC34 cells were grown to 70–80% confluency before transfection with pCI/Neo PGRMC1-3 × HA. Transfection was carried out using Lipofectamine 2000 (Life Science Technology, Grand Island, NY) according to the manufacturer's instruction. Three 15-cm dishes were used for each condition. Control cells were transfected with the same amount of the pCI/neo plasmid that does not contain the PGRMC1 gene. Forty-eight hours post-transfection, culture media were removed; cells were washed twice with ice-cold PBS and then scraped from culture dishes. Cells were pelleted by centrifugation at 500 ×* g* for 5 min and used immediately for membrane preparations.

### 2.8 Preparation of NSC34 and PC12 Cell Membranes

Cell membrane preparation was performed following our established method (Guo et al., 2012). Briefly, cell pellets were suspended in PBS and sonicated on ice using the following settings: 5 sets (10 strokes/set) at duty cycle = 50, output control = 5. Cell homogenates were centrifuged at 8000 ×* g* for 10 min, and the resulting supernatant was collected and subjected to ultracentrifugation at 100,000 ×* g* for 1 h. The supernatant was discarded and the pellet was gently washed twice with ice-cold PBS. The pellet was then resuspended in PBS and sonicated (3 sets, 10 strokes/set at duty cycle = 50, output control = 5) to homogeneity. Protein concentration was then measured using the standard Bradford reagent (Sigma-Aldrich, St. Louis, MO).

### 2.9 Preparation of Rat Liver Membranes

Rat liver membranes, a rich source of sigma-2 receptor, were prepared as described in our previous reports (Pal et al., 2007, Guo et al., 2012) from frozen tissues (Pel Freez Biologicals, Rogers, AR). Liver tissues were homogenized (10 ml buffer/g wet tissue) by 4 bursts of 10 s each using a Brinkman polytron (American Laboratory Trading Inc., East Lyme, CT) on setting 6 in ice-cold sodium phosphate buffer (10 mM pH 7.4) containing 0.32 M sucrose and a cocktail of protease inhibitors (20 μg/ml leupeptin, 5 μg/ml soybean trypsin inhibitor, 100 μM phenylmethylsulfonyl fluoride (PMSF), 100 μM benzamidine and 1 mM EDTA). The membrane suspension after homogenization was centrifuged for 10 min at 17,000 ×* g* and the supernatant was subjected to ultracentrifugation at 100,000 ×* g* for 1 h to collect membrane fractions. The pellet from the second centrifugation was resuspended to a protein concentration of 10 mg/ml in the foregoing buffer, snapped frozen and stored at − 80 °C.

### 2.10 Western Blot Analyses

Protein samples (membranes from Control, PGRMC1 knockout, and PGRMC1 overexpressing NSC34 cells) of 25 μg each were separated on a 12% SDS gel at 140 V. Proteins were transferred to a polyvinyldifluoride (PVDF) membrane (Millipore, Billerica, Massachusett, 0.45 μm) in Tris–Glycine Buffer (25 nM Tris-Base, 200 mM Glycine) at 40 V for 3 h at 4 °C. The PVDF membrane was blocked with 5% non-fat dry milk in TBS containing 0.1% Tween-20 (TBST) for 1 h at room temperature, incubated with a rabbit anti-PGRMC1 antibody (Proteintech, Chicago, IL) overnight at 4 °C, and then washed 3 times for 15 min each in TBST followed by incubation with anti-Rabbit IgG HRP for 1 h at room temperature. After a thorough wash of the membrane the specific PGRMC1 band was illuminated with chemiluminescence reagents (Millipore, Billerica, MA).

### 2.11 Photolabeling With [^125^I]-Iodo-azido-fenpropimorph ([^125^I]-IAF)

The synthesis of [^125^I]-IAF and photolabeling was performed according to our published method (Pal et al., 2007). For photolabeling experiments, each membrane sample of 200 μg proteins was preincubated with 20 μM 1,3-di-o-tolylguanidine (DTG), 5 μM (+)-pentazocine, or various concentrations of CM compounds (Matsumoto et al., 2014) in 50 mM Tris–HCL (pH 7.4) for 30 min at 32 °C. [^125^I]-IAF was then added (final 1 nM) and incubated for another 40 min with gentle shaking in the dark. Photoactivation of [^125^I]-IAF was performed by exposing the samples to a high-pressure AH-6 mercury lamp for 5 s and Laemmli buffer was immediately added to each sample. The samples were then resolved on a 16 × 18 cm SDS gel with 6–18% acrylamide gradient. The gel was dried and exposed to a phosphor screen for 24–48 h and autoradiograms were recorded using a Phosphoimager (Molecular Dynamics).

### 2.12 Assay of Saturation Binding of [^3^H]-1,3-Di-tolylguanidine ([^3^H]-DTG)

Assays were performed according to Hellewell and Bowen (1990). Membranes (28 μg total proteins per reaction) prepared from NSC34 cells of controls, PGRMC1 knockout, or PGRMC1-3 × HA overexpression were incubated in 100 μl 50 mM Tris–HCL (pH 8.0) containing a series of concentrations (3 nm–300 nM) of [^3^H]-DTG for 90 min at 37 °C. The reactions were terminated by rapid filtration through glass fiber filters (Whatman GF/B, Whatman, Maidstone, UK), using a Brandel cell harvester (Brandel, Gaithersburg, MD). The glass fiber filters were pre-soaked in 0.5% polyethyleneimine (Morris et al., 1991) for at least 1 h at room temperature. Filters were washed 4 times with 4 ml of ice-cold 50 mM Tris–HCL, pH 8.0. Radioactivity was quantified in a scintillation liquid (Ultima Gold, PerkinElmer, Waltham, MA) using a liquid scintillation counter (Packard model 1600CA, Packard Instrument Co., Downers Grove, IL).

### 2.13 Inhibition of [^3^H]-DTG Binding to the S2R

Inhibition of [^3^H]-DTG binding to S2R was performed according to Hellewell and Bowen (1990). Briefly, rat liver membranes (28 μg total proteins per reaction) were incubated in 100 μl 50 mM Tris–HCL (pH 8.0) with 60 nM [^3^H]-DTG, 100 nM (+)-pentazocine to selectively mask the sigma-1 receptor binding sites, and a series of concentrations of an inhibitor to be tested. After incubation at 37 °C for 60 min, the reaction was terminated by rapid filtration through glass fiber filters, using a Brandel cell harvester and processed as described above. IC_50_ values were then converted to K_I_ values using the Cheng–Prusoff correction with the following equation K_I_ = IC_50_ / (1 + [L] / K_D_) where [L] is the ligand concentration of the radioactive molecule, and K_D_ of [^3^H]-DTG for the sigma-2 receptor was previously determined to be 60 nM (Hellewell and Bowen, 1990). Nonspecific binding was monitored in the presence of 10 μM *nonradioactive* haloperidol and subtracted from total [^3^H]-DTG binding.

### 2.14 Inhibition of [^3^H]-Progesterone Binding to PGRMC1

Inhibition of [^3^H]-progesterone binding to PGRMC1 was performed according to Peluso et al. (2008). Briefly, rat liver membranes (14 μg total proteins per reaction) were incubated in 100 μl 50 mM Tris–HCL (pH 8.0) with 30 nM [^3^H]-progesterone, 100 nM (+)-pentazocine to selectively mask the sigma-1 receptor binding sites, and a series of concentrations of an inhibitor to be tested. After incubation at 37 °C for 60 min, the reaction was terminated by rapid filtration through glass fiber filters, using a Brandel cell harvester and processed as described above. IC_50_ values were then converted to K_I_ values using the Cheng–Prusoff correction with the following equation K_I_ = IC_50_ / (1 + [L] / K_D_) where [L] is the ligand concentration of the radioactive molecule, and K_D_ of [^3^H]-progesterone for PGRMC1 was previously determined to be 30 nM. Nonspecific binding was monitored in the presence of *nonradioactive* progesterone at 10 μM and subtracted from total [^3^H]-progesterone binding.

### 2.15 Statistical Analysis

Data are presented as mean ± standard error of the mean (SEM). Statistical analysis was conducted using two-tailed unpaired Student's t-test. Data are considered statistically significant when a P value is < 0.05.

## 3 Results

### 3.1 The S2R is not a Splice Variant of PGRMC1

**Fig. 1:**
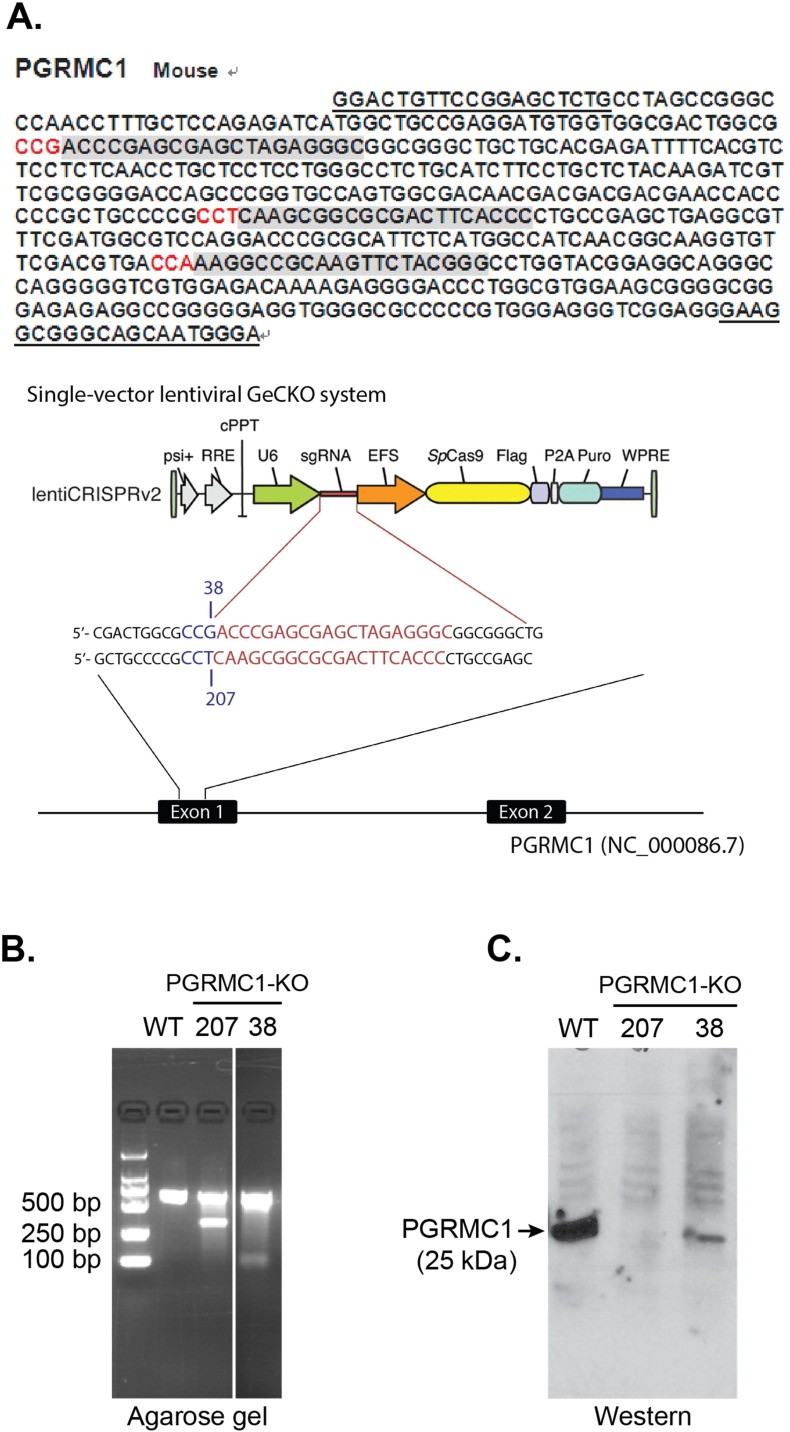
PGRMC1 knockout using a CRISPR/Cas9 approach. A. Schematic of the PGRMC1 sgRNA/Cas9-expressing lentiviral constructs for knocking out PGRMC1. Gray areas show the three candidate sgRNA sequences in the exon-1 of the PGRMC1 gene, of which two were used for the CRISPR/Cas9 constructs. B. A representative DNA gel of control and PGRMC1 knockout (clones 38 and 207) NSC34 cells verifying the Cas9 cleavage of the genomic DNA. Control refers to the NSC34 cells transfected with the control vector expressing Cas9 but not an sgRNA. C. Western blotting detection of PGRMC1 in control and PGRMC1 knockout (clones 38 and 207) NSC34 cells.

The discrepancy in apparent molecular weight on SDS gels between PGRMC1 (25 kDa) and the S2R (18–21 kDa) (Hellewell and Bowen, 1990, Pal et al., 2007) prompted us to determine whether the S2R is a splice variant of PGRMC1. In order to address this question, we constructed a PGRMC1 knockout cell line using a CRISPR/Cas9 system (Fig. 1A). Application of this new genome-editing technology is rapidly expanding because of its effectiveness in completely depleting an endogenous protein (Doudna and Charpentier, 2014), especially in contrast to siRNA knockdown, which is able to reduce a targeted protein typically by 50–60%. Indeed, the two PGRMC1 knockout clones showed complete and ~ 90% deletion of PGRMC1 protein, respectively, as estimated by Western blotting (Fig. 1C). We then isolated membranes from the wild-type control and knockout NSC34 cells and performed [^125^I]-IAF photolabeling experiments following our previously published method (Pal et al., 2007).

**Fig. 2:**
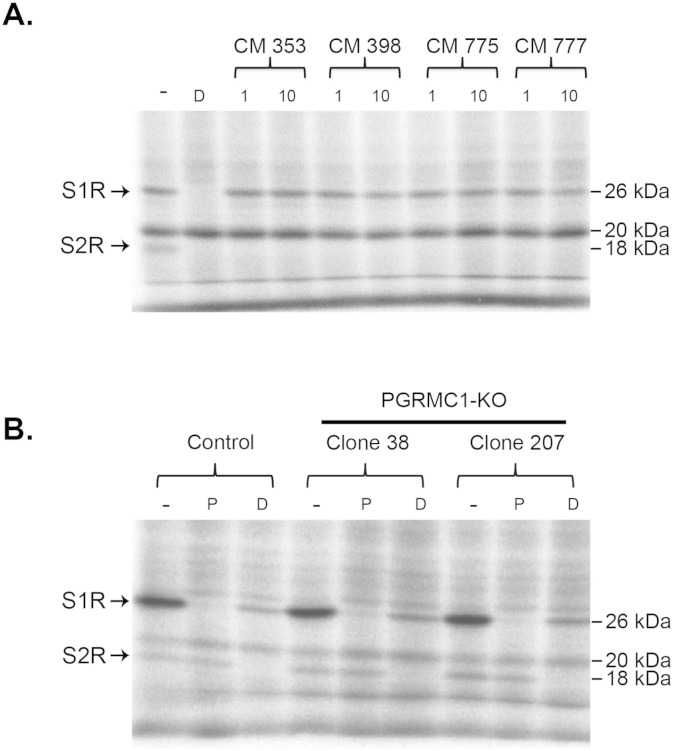
[^125^I]-IAF photolabeling of the S2R is protected by S2R-specific ligands but not affected by PGRMC1 knockout. A. [^125^I]-IAF photolabeling of the S2R in PC12 membranes is protectable by CM compounds. [^125^I]-IAF photolabeling of both S1R and S2R was protected by 20 μM DTG (D) while CM compounds (CM 353, CM 398, CM 775, and CM 777, see Fig. S1 for affinities to sigma receptors) (Matsumoto et al., 2014) selectively blocked the labeling of the S2R but not the S1R. Two concentrations (1 and 10 μM) were used for CM compounds. B. [^125^I]-IAF photolabeling of the S2R in membranes prepared from control and PGRMC1 knockout (clones 38 and 207) NSC34 cells. 5 μM (+)-pentazocine (P) protected against [^125^I]-IAF photolabeling of the S1R while 20 μM DTG (D) protected against photolabeling of both the S1R and the S2R. Note that the background photolabeled bands were not protected by these specific sigma ligands.

Because of the lack of a known S2R amino acid sequence, photo-affinity labeling has remained the most powerful approach to visualize this receptor on a SDS gel (Hellewell and Bowen, 1990, Hellewell et al., 1994, Pal et al., 2007). The basic principle is to covalently crosslink a photoactivatable S2R-binding probe to the receptor such that the probe (radioactive- or fluorescent-labeled) remains with the protein even after denaturation with SDS. It is well known that the S2R and S1R bind DTG with similar high affinities (~ 50 nM) but the S2R does not bind (+)-pentazocine which is a high-affinity ligand for the S1R (< 10 nM) (Hellewell et al., 1994), thus these two sigma receptors can be readily distinguished. Autoradiography showed an [^125^I]-IAF photolabeled band of S1R at 26 kDa which was protected by (+)-pentazocine and DTG (Fig. 2). The S2R band appeared at ~ 18 kDa and was protected by DTG but not by (+)-pentazocine (Fig. 2, A and B, see Fig. S1 for chemical structures of ligands). We further tested the specificity of the [^125^I]-IAF photoprobe using a group of ligands that possess superior binding affinity as well as selectivity towards the S2R (Fig. S1) (Matsumoto et al., 2014), e.g*.* CM398 has a K_I_ of 0.43 nM for the S2R in contrast to a K_I_ of 560 nM for the S1R, as determined previously in competitive [^3^H]-DTG binding assays (Matsumoto et al., 2014). An n-isothiocyanate derivative (CM572) has been recently reported as an irreversible selective affinity label for the S2R (Nicholson et al., 2015). As shown in Fig. 2B, these CM compounds at 1 μM completely blocked photolabeling of the S2R without reducing the S1R labeling intensity, further validating [^125^I]-IAF photolabeling as a highly specific approach for detection of the S2R. It is noteworthy that an unidentified protein of ~ 20 kDa was also photolabeled, but the labeling was not protected by any of the foregoing S1R and S2R ligands (Fig. 2). This result provides another line of evidence for the specificity of [^125^I]-IAF photolabeling of sigma receptors.

Most interestingly, the comparison of [^125^I]-IAF photolabeling between control and two PGRMC1 knockout clones showed two clear results. First, the photolabeled S2R band was not diminished in the knockout clones compared to the control. Second, there was not a DTG-protectable photolabeled band consistent with endogenous PGRMC1 (25 kDa) since the only two DTG-protectable bands were the S1R and the S2R (Fig. 2A). While the S1R also migrated at ~ 25 kDa, this photolabeled band was readily protected by (+)-pentazocine. (+)-Pentazocine is not a ligand for PGRMC1, as indicated in Xu et al. (Xu et al., 2011), thus precluding the possibility of this prominent band being PGRMC1. Moreover, a lack of influence of PGRMC1 knockout on photolabeling of the S2R indicated that the existence of a S2R ligand-binding pocket formed by a PGRMC1/S2R complex is unlikely. Taken together, these results demonstrate that the S2R is not a splice variant of PGRMC1 and thus these two proteins are derived from different genes.

### 3.2 Neither Knockout nor Overexpression of PGRMC1 Altered [^3^H]-DTG/S2R Binding Characteristics

**Fig. 3:**
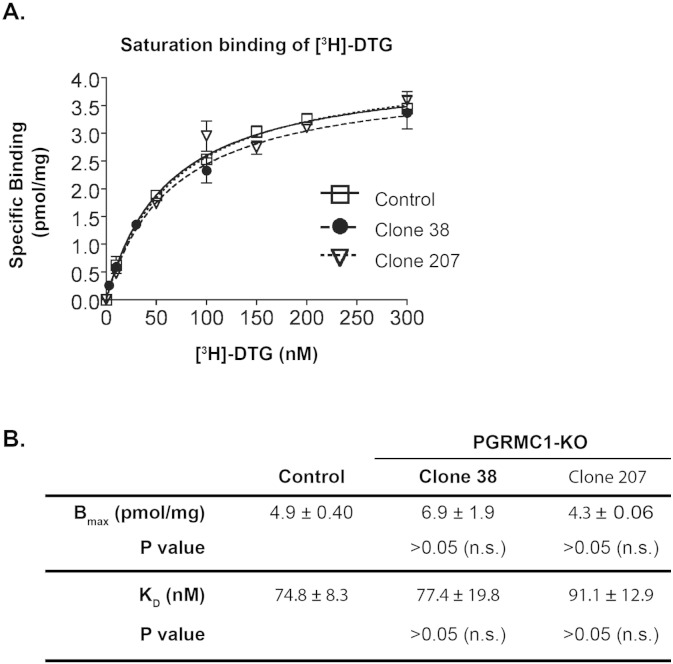
Eliminating PGRMC1 protein does not alter [^3^H]-DTG binding to the S2R in cell membranes. A. A representative of [^3^H]-DTG saturation binding in membranes prepared from control and PGRMC1 knockout (clones 38 and 207) NSC34 cells, (+)-pentazocine (100 nM) was included to mask [^3^H]-DTG binding to the S1R such that [^3^H]-DTG would be bound only to the S2R and measured as specific S2R binding. Nonspecific binding was measured (by adding haloperidol) and subtracted. Control refers to the NSC34 cells transfected with the control vector for the expression of Cas9 but not an sgRNA. B. Statistics. Maximum binding (B_max_) and equilibrium dissociation constants (K_D_) for [^3^H]-DTG were calculated using a Prizm software and reported as mean ± SEM of three separate experiments each performed in triplicates. Not significant (n.s.).

While the foregoing data indicated that PGRMC1 knockout did not affect [^125^I]-IAF photolabeling of the S2R, we cautioned that, alternatively, PGRMC1 may be another DTG-binding protein that does not bind the photoprobe [^125^I]-IAF. To test a correlation between PGRMC1 expression levels and [^3^H]-DTG binding, we performed [^3^H]-DTG saturation binding assays using membranes prepared from wild type control and PGRMC1 knockout cells. Thus far the only known high-affinity DTG binding sites are the S2R and S1R, both with K_D_ values for DTG in a range of ~ 20–80 nM (depending on sources of membranes and assay methods) (Weber et al., 1986, Quirion et al., 1992, Itzhak and Kassim, 1990, Itzhak, 1987). Therefore the accepted radioligand binding assays for the S2R involve using [^3^H]-DTG in the presence of 100 nM (+)-pentazocine to mask the S1R binding site (Quirion et al., 1992). As shown in Fig. 3A, [^3^H]-DTG binding curves of the control and two knockout clones were almost identical. Statistical analyses indicated that neither B_max_ nor K_D_ was significantly different between control and knockout, which holds true for both of the PGRMC1 knockout clones (Fig. 3B). Thus, these results demonstrate that DTG binding (to the S2R) characteristics in cell membranes do not correlate with PGRMC1 protein levels. A disconnect between the S2R and PGRMC1 was also evidenced in our preliminary studies (Ruoho et al., 2013, Chu et al., 2015) as well as a new report by Abate et al. using MCF-7 cells (Abate et al., 2015).

**Fig. 4:**
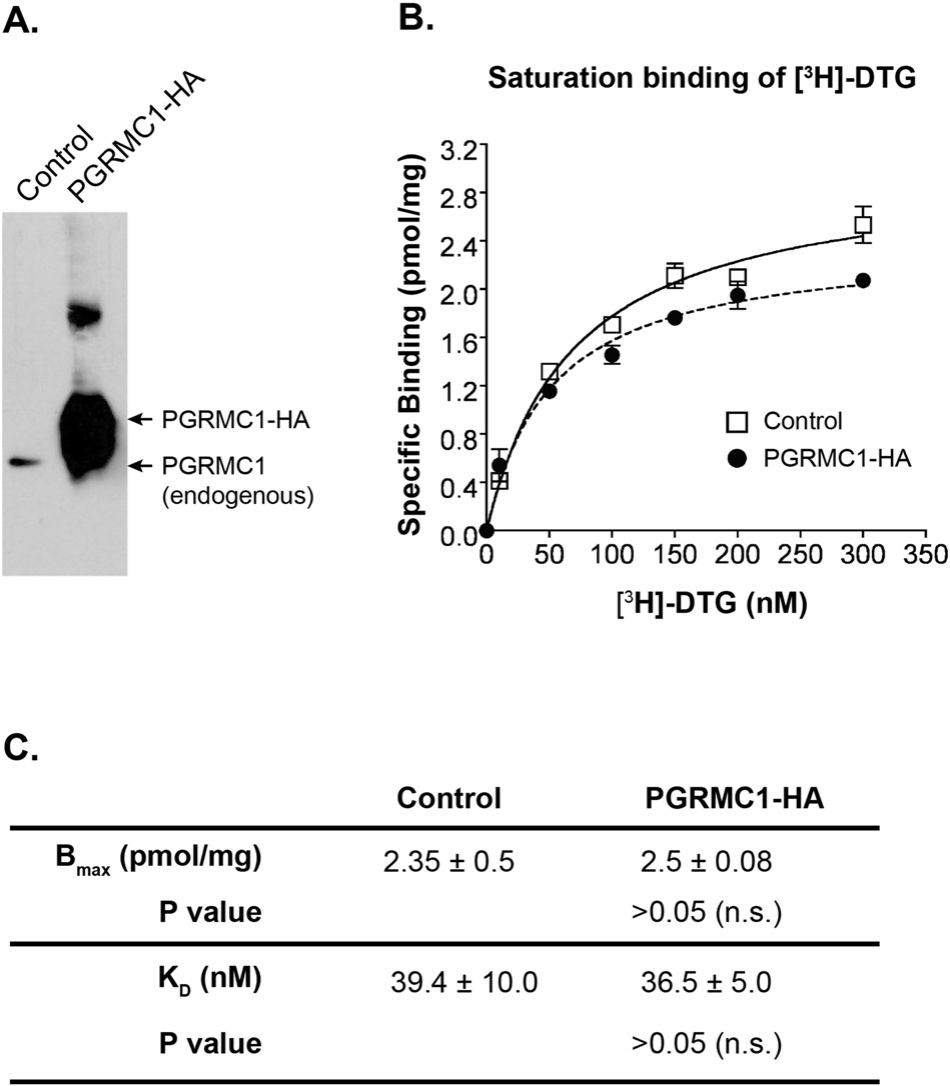
PGRMC1 overexpression does not change [^3^H]-DTG binding to the S2R in cell membranes. A. A representative Western blot detecting PGRMC1 in membranes prepared from control and PGRMC1-3 × HA overexpressing NSC34 cells. Control refers to the NSC34 cells transfected with the same expression vector but without the PGRMC1 gene. B. A representative experiment of [^3^H]-DTG saturation binding (as described in Fig. 3A) in membranes prepared from control and PGRMC1-3 × HA overexpressing NSC34 cells. C. Statistics. Maximum binding (B_max_) and equilibrium dissociation constants (K_D_) for [^3^H]-DTG were calculated using a Prizm software and reported as mean ± SEM of three separate experiments each performed in triplicates. Not significant (n.s.).

Furthermore, we also performed [^3^H]-DTG saturation binding assays in a gain-of-function experiment using PGRMC1-HA over-expressed in NSC34 cells. We infer that if PGRMC1 were a high-affinity DTG binding site, elevation of PGRMC1 protein levels would increase maximum [^3^H]-DTG binding. Remarkably, our data show that neither the B_max_ nor K_D_ changed significantly in response to PGRMC1 overexpression (Fig. 4). These results further confirm a lack of correlation between PGRMC1 and high affinity [^3^H]-DTG binding to the S2R.

### 3.3 PGRMC1 is not a High-affinity DTG Binding Site

Xu et al*.* showed that DTG at 1 μM protected the photolabeling of PGRMC1 (with WC-21) (Xu et al., 2011), however the affinity of DTG for PGRMC1 was not reported. To directly address whether DTG binds to PGRMC1 with high affinity, we performed competitive binding assays with a fixed low concentration of [^3^H]-progesterone (30 nM) and increasing concentrations of unlabeled DTG. Progesterone is known to be a high-affinity (K_D_ = 35 nM) ligand for PGRMC1 (Peluso et al., 2008). At 30 nM, the S2R is not expected to bind progesterone because the K_I_ of progesterone for the S2R is 14.2 ± 4.9 μM in rat liver membranes (Fig. 6). Thus, high-affinity [^3^H]-progesterone binding serves as a discriminative tool for the detection of PGRMC1, providing a unique opportunity for us to assess the DTG and haloperidol binding affinities for PGRMC1.

**Fig. 5:**
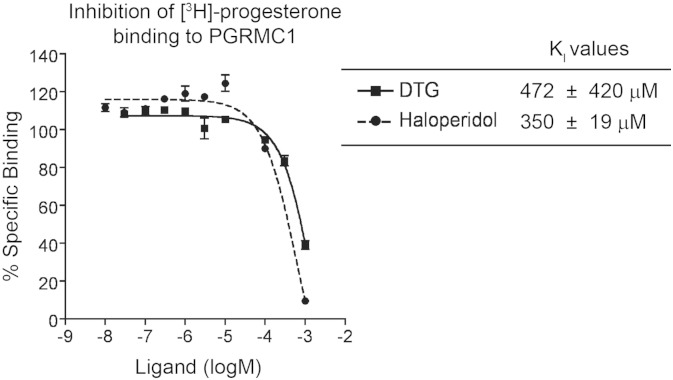
Assessment of DTG and haloperidol binding affinities for PGRMC1. Experiments were performed with rat liver membranes. Shown on the left is a representative inhibition curve of [^3^H]-progesterone binding (at 30 nM) to PGRMC1 in the presence of increasing concentrations of non-radioactive DTG or haloperidol. Nonspecific binding was measured by the addition of 10 μM of nonradioactive progesterone and subtracted, as previously described by Peluso et al. (2008). Inhibition constants (K_I_) are reported as mean ± SEM from three separate experiments each performed in triplicates for DTG and two separate experiments each performed in triplicates for haloperidol.

**Fig. 6:**
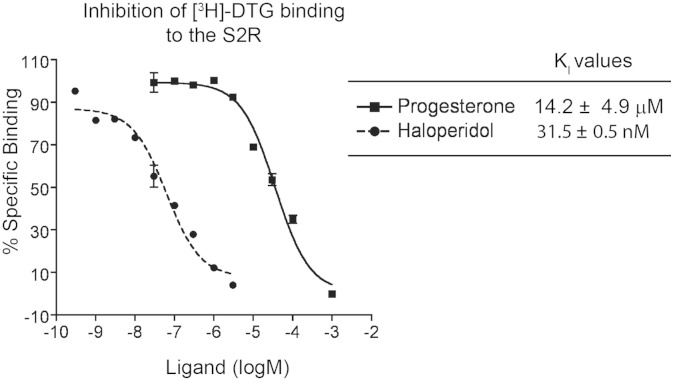
Assessment of progesterone and haloperidol binding affinities for the S2R. Experiments were performed with rat liver membranes. Shown on the left is a representative inhibition curve of [^3^H]-DTG binding (at 60 nM) to the S2R in the presence of increasing concentrations of non-radioactive progesterone or haloperidol. Nonspecific binding was measured by the addition of 10 μM of nonradioactive haloperidol and subtracted. Inhibition constants (K_I_) are reported as mean ± SEM from 3 separate experiments each performed in triplicates.

As shown in Fig. 5, the K_I_ of DTG and haloperidol for PGRMC1 in rat liver membranes were determined to be 472 ± 420 μM and 350 ± 19 μM, respectively. In stark contrast, the affinities of DTG (20–80 nM) (Vilner et al., 1995, Hellewell et al., 1994, Matsumoto et al., 2014) and haloperidol (31.5 ± 0.5 nM, Fig. 6) for the S2R are several orders of magnitude higher in the same (rat liver) membranes further supporting a lack of correlation between PGRMC1 and the S2R binding site. Conversely, progesterone, a classic high-affinity (K_D_ = 35 nM) ligand of PGRMC1 (Peluso et al., 2008), showed a very low affinity for the S2R in rat liver membranes (K_I_ = 14.2 ± 4.9 μM, Fig. 6). Thus, these results present further compelling evidence for the conclusion that PGRMC1 and the S2R are two different binding sites.

## 4 Discussion

As a potential target for tumor diagnosis and treatment, the S2R has been the subject of many studies, but its identity has remained a mystery. While PGRMC1 was recently reported to be the S2R (Xu et al., 2011), inconsistency remains in their key characteristics. In order to resolve the ambiguity of genetic and pharmacological relationships between PGRMC1 and the S2R, we first determined whether PGRMC1 and the S2R are derived from the same gene. Through CRISPR/Cas9-mediated genome-editing (Cong et al., 2013), we knocked out PGRMC1 in a motor neuron-like cell line (NSC34). We found that [^125^I]-IAF photolabeling of the S2R and [^3^H]-DTG/S2R binding characteristics remained unchanged in the PGRMC1 knockout versus the wild type control. These data indicate that PGRMC1 and S2R are genetically two different proteins. Second, we determined the inhibition constant (K_I_) of DTG and haloperidol for binding to PGRMC1 using [^3^H]-progesterone at its reported K_D_ of 35 nM for PGRMC1 (Peluso et al., 2008). The affinities of DTG and haloperidol for PGRMC1 in rat liver membranes were found to be 472 ± 420 μM and 350 ± 19 μM, respectively. These DTG and haloperidol K_I_ values for PGRMC1 are > 3 orders of magnitude higher than their K_I_ values for the S2R in the same membranes (20–80 nM) (Vilner et al., 1995, Hellewell et al., 1994, Matsumoto et al., 2014). This striking difference indicates that PGRMC1 does not have the signature pharmacological characteristics that have been associated with the S2R in the literature (i.e*.* tight binding to DTG and haloperidol) (Bowen et al., 1989, Hellewell and Bowen, 1990, Hellewell et al., 1994). Therefore, PGRMC1 is a non-S2R binding site in mammalian tissues.

Based on the original discoveries, the S2R has been authentically defined as a high-affinity DTG binding site with a molecular weight at 18–21 kDa (Bowen et al., 1989, Hellewell and Bowen, 1990, Hellewell et al., 1994, Itzhak, 1987, Weber et al., 1986). According to this definition, the results presented here indicate that PGRMC1 is not the true, long sought-after S2R. This conclusion is supported by several lines of evidence: 1) PGRMC1 is a 25 kDa protein whereas the S2R has been determined to be 18–21 kDa by photolabeling (Hellewell and Bowen, 1990, Hellewell et al., 1994, Pal et al., 2007). 2) PGRMC1 knockout did not reduce [^125^I]-IAF photolabeling of the S2R (18–21 kDa band) that was protectable by DTG and the highly S2R-selective CM compounds. 3) Either knockout or overexpression of PGRMC1 did not significantly alter [^3^H]-DTG binding to the S2R in NSC34 cell membranes (Fig. 3) or HEK293 cell membranes (Abate et al., 2015). These lines of evidence demonstrate that PGRMC1 and the S2R are expressed by different genes. 4) Most importantly, the DTG (and haloperidol) affinity for PGRMC1 is more than three orders of magnitude lower than that determined for the S2R in rat liver membranes, indicating that PGRMC1 is not the S2R ligand binding site. Thus, taken together previous studies and the data presented herewith, it is premature to equate PGRMC1 to the S2R (Izzo et al., 2014). Further research is needed to discover the S2R sequence followed by careful biochemical, pharmacological and functional verifications.

In summary, through genome editing in combination with chemical biology uniquely tailored for characterization of the S2R, we have addressed the questions critically important with regard to the true identity of the S2R. We found that (1) PGRMC1 and the S2R are derived from different genes; (2) PGRMC1 is not a high-affinity DTG and haloperidol binding site. In others words, PGRMC1 is not the originally defined S2R. This clarification may impact the research community as follows: 1) Since the S2R has been found to be functionally important in cancer and neuronal diseases (Cassano et al., 2006, Crawford and Bowen, 2002, Kashiwagi et al., 2009, Vilner and Bowen, 2000, Vilner et al., 1995, Wheeler et al., 2000), the findings reported herein pose a timely reminder that the true, high-affinity DTG binding S2R has yet to be cloned. 2) Our study will help clarify confounding messages resulting from the premature conclusion that PGRMC1 is the S2R (Xu et al., 2011). For example, a pro-autophagy function has recently been attributed to the S2R, based on studies on PGRMC1 (Mir et al., 2013), but whether the true S2R is responsible for autophagy or not remains unknown. 3) Recognizing that PGRMC1 and the S2R are different binding sites is critical for rational drug design and development of therapeutic molecules, since targeting PGRMC1 may not necessarily produce the same functional outcomes as targeting the S2R in animal models or human patients. Thus, the results presented here underscore the necessity of more careful studies on the S2R and an urgent need to decode the sequence of this intriguing receptor.

## Conflicts of Interest

The authors declare no conflicts of interest.

## Funding

This research was supported by UWSMPHPRJ82KR to AER, NIH grant EY022678 and Morgridge Institute for Research and the James Christenson Estate Macular Degeneration Research Fund (UDDS 531800) to L-WG, NIH P30 grant EY016665 to the University of Wisconsin Madison Vision CORE, and NIH Grants DA023205 and GM104932 to CMcC.
