# Supplementary material for: The Sigma-2 Receptor and Progesterone Receptor Membrane Component 1 are Different Binding Sites Derived From Independent Genes
Source: eBioMedicine. 2015 Oct 19;2(11):1806–13. doi: 10.1016/j.ebiom.2015.10.017 (PMC4740303; doi:10.1016/j.ebiom.2015.10.017)
Supplement: Supplementary file 1 — Supplementary material. [file mmc1.docx]

**The Sigma-2 Receptor and PGRMC1 are Different Binding Sites Derived from Independent Genes**

Uyen B. Chu^1,*^, Timur A. Mavlyutov^2,*^, Ming-Liang Chu^2^, Huan Yang^2^, Amanda Schulman^1^, Christophe Mesangeau^4^, Christopher R. McCurdy^4^, Lian-Wang Guo^2,3^, and Arnold E. Ruoho^1^

^1^Department of Neuroscience,

^2^Department of Surgery,

^3^McPherson Eye Research Institute,

University of Wisconsin School of Medicine and Public Health, Madison, Wisconsin

^4^Department of BioMolecular Sciences, School of Pharmacy, University of Mississippi

^*^Uyen B. Chu and Timur A. Mavlyutov are co-first authors

Short title: ***PGRMC1 is not the true Sigma-2 receptor***

To whom correspondence should be addressed:

Arnold E. Ruoho, Professor

Department of Neuroscience

University of Wisconsin School of Medicine and Public Health

1300 University Avenue, Madison, WI 53706

Tel. (608) 263-5382

Fax. (608) 265-4493

Email: [aeruoho@wisc.edu](mailto:aeruoho@wisc.edu)

Or

Lian-Wang Guo, Assistant Professor

Department of Surgery

University of Wisconsin School of Medicine and Public Health

1111 Highland Avenue, Madison, WI 53705

Tel. (608) 262-6269

Email: [guo@surgery.wisc.edu](mailto:guo@surgery.wisc.edu)

**Supplemental Figure:**

**
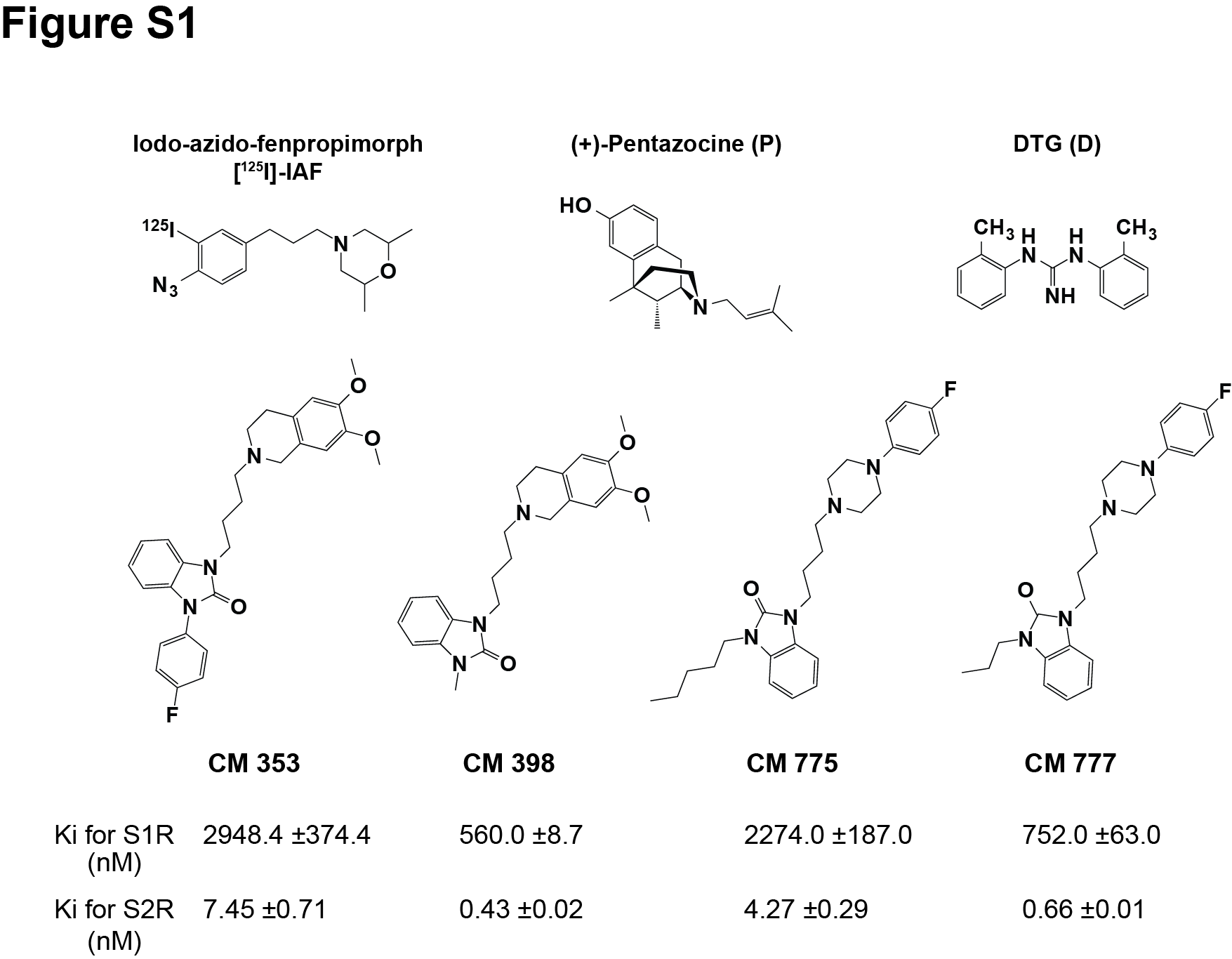
**

**Figure S1: Chemical structures of S1R and S2R ligands**

Kis of the CM compounds[^1^](#_ENREF_1) are presented under their respective structures, indicating a high selectivity for the S2R

1. Matsumoto RR, Nguyen L, Kaushal N, Robson MJ. Sigma (sigma) receptors as potential therapeutic targets to mitigate psychostimulant effects. *Adv Pharmacol*. 2014;69:323-386
